# Navigating Complexities In Randomized Trials: Insights From The EMERGE Trial Amidst Pandemic And Cybersecurity Threat

**DOI:** 10.12688/hrbopenres.14233.1

**Published:** 2025-09-10

**Authors:** Christine Newman, Fidelma Dunne, Alberto Alvarez-Iglesias, Marie Browne, Michelle Courcy Byrnes, Declan Devane, Paddy Gillespie, Chloe Ryan, Roberta Scairati, Sinead Wallace, Martin O'Donnell, Andrew Smyth

**Affiliations:** 1 College of nursing, medicine and health science, University of Galway, Galway, County Galway, Ireland; 2 HRB Clinical Research Facility, Galway, County Galway, Ireland; 3 Galway University Hospital, Co Galway, Ireland; 4 University of Galway, HRB Trials Methodology Network, Galway, Ireland, Ireland; 5 School of Nursing and Midwifery, University of Galway, Galway, County Galway, Ireland; 6 School of Business and Economics, University of Galway, Galway, County Galway, Ireland

**Keywords:** COVID-19; Diabetes, gestational; Clinical Trials; Computer security

## Abstract

**Introduction:**

The COVID-19 pandemic caused by the SARS-CoV-2 virus changed the global landscape of clinical trials; changes in staffing, clinical priorities and recruitment challenges led to the delay or termination of many clinical trials. The Early Metformin in Gestational Diabetes (EMERGE) trial was completed despite COVID-19 and institutional computer network attacks (CNAs).

**Methods:**

Here we outline the challenges encountered and the solutions employed to continue the active recruitment of a potentially high risk patient cohort during the Covid-19 pandemic and detail the techniques adapted to enable recruitment, adverse event reporting and lab reporting during two critical computer network attacks. We present our solutions from both the site and sponsor perspective.

**Results:**

COVID-19 and the computer network attacks presented many challenges for the clinical trial site and Sponsor including staff shortages, remote working, deferred recruitment, changes in the diagnostic pathway for gestational diabetes mellitus and pivoting to remote working and telemedicine. The first CNA impeded clinical and laboratory systems, impacting recruitment and follow up. The second CNA delayed access to the Clinical Data Management System. Solutions employed included telemedicine, videoconferencing, shortened in-person reviews, contact tracing, COVID-19 risk mitigations, paper-based back-up systems and vigorous data integrity checks.

**Conclusion:**

Overcoming the multiple challenges posed by COVID-19 and computer network attacks required rapid re-organisation, teamwork and flexibility from staff and Sponsor. The use of both digital solutions and paper-based records highlighted the importance of rapid innovation and comprehensive protocol planning.

## Introduction

The COVID-19 global pandemic presented unprecedented challenges to government, societal, educational, and healthcare organisations, particularly during 2020. Overnight, organisations were required to pause completely or implement significant changes to the delivery of services and organisation of staff for business continuity. Healthcare was impacted heavily due to public healthcare needs and caring for individuals with COVID-19). At the same time, much of the world pivoted to remote working practices. The most notable changes in healthcare were altered staffing ratios, increased personal protective equipment, a sharp increase in virtual clinical review (telephone or videoconferencing) and a surge in healthcare needs by critically unwell patients with SARS-CoV-2.

In addition to routine clinical care, ongoing clinical trials faced significant challenges. While many institutions (including Academic Sponsors, Industry Sponsors and Funders) made allowances for public health restrictions limiting research (through grant and deadline extensions), many non-SARS-CoV-2 trials were deferred or terminated, including some in their final stages ^1^. Although recruitment for many trials was paused temporarily due to public health uncertainties, it remained crucial to ensure that active trial participants received follow-up, protocol compliance and supply of investigational-medicinal products (IMP), despite challenges brought about by travel restrictions, limited hospital attendance, staff shortages and the prioritisation of COVID-19 trials. International clinical trials faced challenges due to varying public health restrictions, and government approaches across jurisdictions, making it difficult to standardise trial execution.

COVID-19-induced changes in healthcare delivery and the clinical research landscape led to well-documented effects on clinical trials. In both Europe and the United States, the number of non-Covid related studies dropped and didn’t recover until 2021 ^2^. There were concerns about the completeness of data from studies conducted during this period ^3, 4^. Meanwhile, clinical research infrastructure pivoted to prioritising COVID-19 research. Therefore, many new trials initiated during 2020 focused on COVID-19, further impacting non-COVID-19 research. In our jurisdiction, there were additional impacts from increased cyberattacks ^5, 6^.

Despite these significant challenges, we successfully recruited 535 participants with gestational diabetes mellitus (GDM) into the Early Metformin in Gestational Diabetes (EMERGE) trial. The details of the EMERGE trial have been published previously ^7, 8^. In brief, the EMERGE trial was a double-blind, placebo-controlled, multi-centre, two-parallel-group randomised controlled trial (RCT), where women with GDM were randomised to metformin or placebo, in addition to standard nutritional and lifestyle advice. The primary outcome was a composite of insulin initiation and fasting plasma glucose ≥5.1 mmol/L at 32 or 38 weeks’ gestation ^8^. Here, we discuss the challenges of COVID-19 and institutional computer network attacks (CNA) that we navigated.

## Methods

Key staff as identified by the principal investigator (PI) involved in the design, execution and oversight of the EMERGE trial, including trial site representatives, investigators, sub-investigators, Sponsor delegates and the Trial Working Group including trial co-ordinator were invited to discuss their perspectives of the challenges posed by the Covid-19 pandemic and CNAs. All contributors are listed as authors. All invited staff agreed to participate, and provided written informed consent. Ethical approval was granted by the Ethics Review Board in Galway University Hospital (CA 3158). The discussions, held by videoconferencing, were conducted by two researchers - FD (PhD and PI) and CN (MD and sub-PI). All study participants were known to both interviewers from previous work on the trial. Prior to the discussion CN and FD decided on key questions/themes to be discussed and new themes introduced by the participants were explored during the interview where relevant ^9^.

## Results

### Clinical trial site challenges from COVID-19

As COVID-19 was declared a global pandemic and emergency, our Institutions agreed to temporarily pause the recruitment of new participants into clinical trials to minimise risks to healthcare staff, research staff and patients. In tandem, research staff were encouraged to work remotely through secure access to Institutional systems. In particular, all non-patient-facing staff (trial coordinator, data manager, pharmacovigilance officer, biostatistician, etc.) transitioned to exclusive remote working. Patient-facing staff (including medical staff, research nurses, research assistants, and clinical trial pharmacists) minimised time they spent on-site and in direct contact with other healthcare staff, trial participants, and patients. In addition, some staff were temporarily redeployed to support COVID-19 testing centres and contact tracing, and there was a temporary moratorium on recruiting new staff. At all times, a sufficient complement of trained and delegated research staff maintained contact with EMERGE active trial participants, but there were delays to recruitment and data query resolution. As our trial participants continued to attend antenatal services, they continued to be closely monitored by obstetricians and midwives. Although aspects of routine obstetric care at our centre were disrupted or shifted to virtual appointments, minimised in-person attendance continued for essential assessments (including blood pressure, urinary, and ultrasound monitoring).

Laboratory staffing was modified to facilitate COVID-19 testing, causing delays in processing biospecimens However, samples were appropriately stored and analysed, ensuring data integrity. In addition, as EMERGE included metformin, which has an established, extensive and excellent safety profile, there were no risks to the safety of trial participants due to these delays in sample processing.

**Figure 1.**
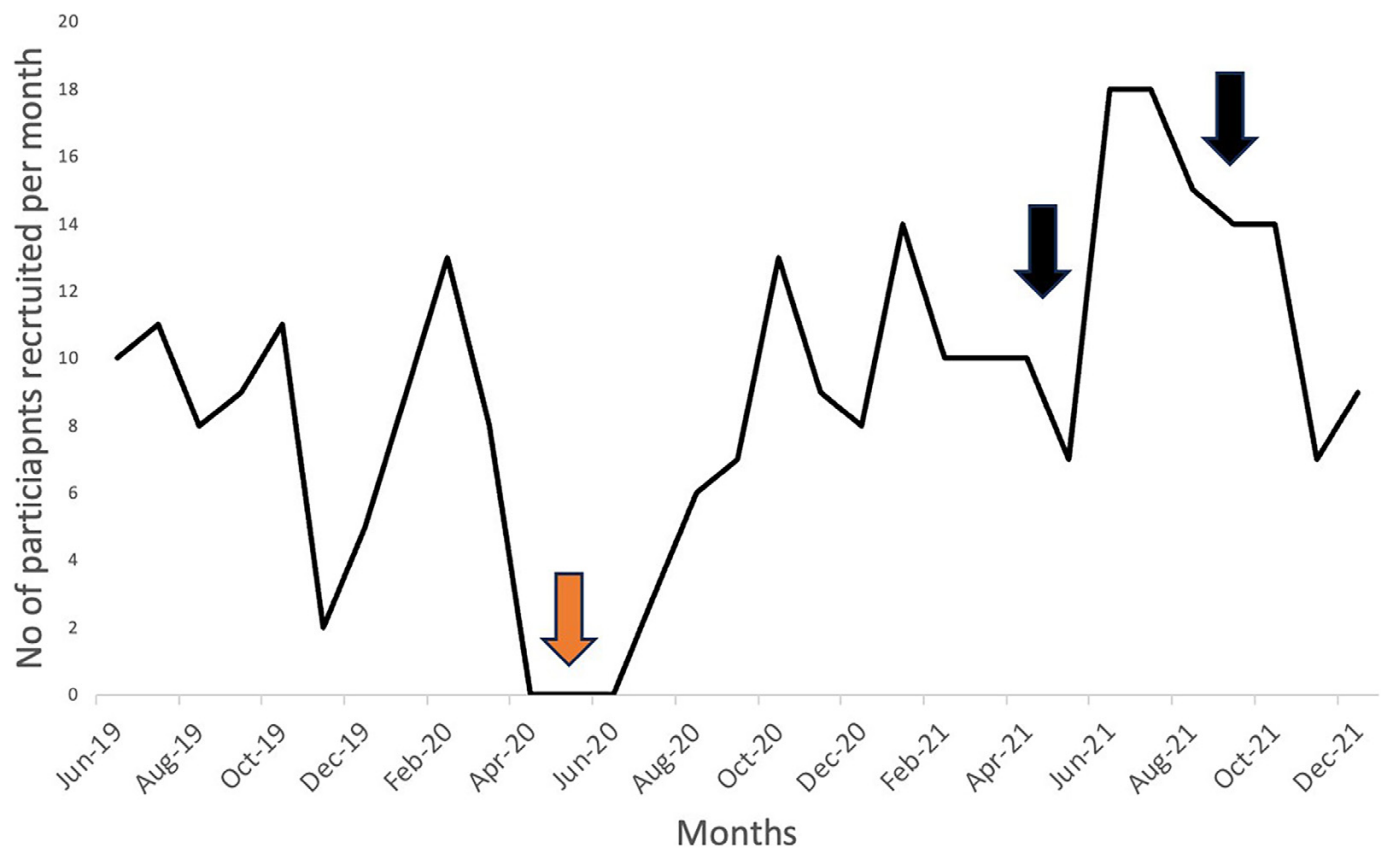
Selected Monthly Participant Recruitment in the EMERGE trial. EMERGE trial recruitment commenced in June 2017, with data shown for 6 months before onset of pandemic to end of 2021; trial recruitment completed in September 2022. Red arrow - pause in recruitment from April-June 2020 (inclusive). Black arrows - indicates CNAs in hospital and university networks

An extensive COVID-19-specific risk assessment (involving the trial site, research centre and Sponsor) was performed, and solutions were implemented to reduce or mitigate risks to staff and participants. This included: (i) telephone and/or videoconferencing facilities to obtain trial data from participants; and (ii) a strict minimisation of in-person contact between participants and staff (including personal protective equipment (PPE)) to strictly necessary activities (e.g. clinical measurement, blood sampling, etc.). This subsequently permitted trial recruitment to resume (four months after the onset of the pandemic). However, it remained limited due to restrictions on the number of individuals permitted to work on-site and number of participants permitted to attend our research centre simultaneously ( Figure 1).

Modifications to routine obstetric care limited the ability of the study team to meet potential new trial participants before 28 weeks and six days gestation (our cut-off for trial eligibility). There were temporary modifications to the GDM diagnostic pathway to reduce patient exposure to healthcare settings and minimise infection spread- the standard 75g oral glucose tolerance test (OGTT) was replaced by a combination of fasting glucose and glycosylated haemoglobin A1c (HbA1c) ^10^. Only women with a previous GDM diagnosis completed a 75g OGTT. Therefore, some patients were diagnosed clinically with GDM but did not satisfy our inclusion criteria (requiring a 75g OGTT with fasting, 1-hour and 2hour glucose levels) and were not randomised into EMERGE. Lastly, routine foetal wellbeing scans were limited during the initial period of the pandemic, which also impacted recruitment as potential participants did not complete ultrasound scans during the first trimester. As our exclusion criteria included <10 ^th^ centile for any dimension, the limited availability of foetal measurement data made such participants ineligible for trial recruitment.

Concurrently, healthcare-related patient anxiety was extremely high. As clinical trial participation was deemed non-essential, routine clinical discussions between obstetrics, diabetes services, and patients often did not incorporate the EMERGE trial during the early stages of the pandemic. Potential participants were also concerned that trial participation would significantly increase in-person healthcare contact and, therefore, additional potential COVID-19 exposure. We reassured participants of the mitigating strategies implemented (including PPE, minimised in-person contact, and alignment with routine care). During trial follow-up, many participants contracted or were close contacts of COVID-19 cases, limiting their ability to attend in-person visits. This included the baseline randomisation visit for some, meaning they could not be enrolled and randomised into the EMERGE trial.

We identified potential participants at an early gestational age by reviewing case notes, including women with previous GDM. Written and verbal trial information was provided by telephone or videoconferencing to reassure and allay fears. As before the pandemic, potential participants were reassured that metformin is not an entirely experimental treatment (e.g., the NICE guidelines in the United Kingdom recommend metformin as a first-line therapy in GDM if lifestyle and nutritional interventions fail to control blood glucose) ^11^. The uniqueness of the EMERGE trial lay in the early use of metformin in pregnancy impacted by GDM. The focus became a more personal interaction between our clinical site team and potential participants by telephone or videoconferencing to reassure and allay fears. The site team also remotely reached out to consultant obstetricians, midwives and other healthcare professionals involved in routine obstetric care to highlight the potential benefits of the EMERGE trial and educate them on mitigating strategies to minimise COVID-19 exposure for trial participants. Potential trial participants were invited to attend a separate antenatal clinic (with reduced numbers compared to routine antenatal clinic) to receive study information and obtain site team members' contact details for additional contact.

 To maximise trial retention, we ensured that study visits were scheduled in line with the standard of care clinic visits whilst also providing flexibility and consideration of practical issues experienced by individual participants. In-person visits between trial participants and site team members were conducted in the same area of the hospital as the routine clinical visit to minimise travel through the hospital setting. Attendance logs were maintained (to facilitate contact tracing), participants did not encounter other participants during study visits, and equipment was thoroughly cleaned between participants. Close and regular communication between trial participants and the site team maximised compliance with required study processes and procedures. No protocol amendment was needed to facilitate telephone-based study visits, as we had built-in flexibility during the design phase of EMERGE. In-person visits were limited to drug dispensing, clinical measurements, or blood draws. For participants who did not speak English as their first language, professional translational services were employed through video or teleconferencing, replacing the in-person requirement for translators (including family members).

## Sponsor perspective

As is typical of regulated clinical trials, the Sponsor maintained close contact with the clinical trial site throughout the EMERGE trial. This was heightened further during the declaration of the public health emergency and the COVID-19 pandemic, as public health guidelines and Institutional decisions rapidly evolved. An extensive COVID-19-specific risk assessment (involving the trial site, research centre and Sponsor) was completed, and solutions were implemented to mitigate risks to staff and participants. The EMERGE trial Sponsor issued advice and direction to the trial site in line with contemporary guidance provided by the Competent Authority and the European Medicines Agency. Initially, our institution temporarily closed recruitment of new participants to trials and ceased in-person study visits, in line with public health guidance. Exceptions were made after the Sponsor agreed that site staff would liaise with patients in the active treatment phase of the trial to maintain an adequate supply of investigational medicinal product (IMP). This included but was not limited to, meeting participants in outdoor car parks or other well-ventilated locations with contactless and witnessed dispensing and collection of EMERGE IMP. Follow-up visits were conducted via telephone to capture data on drug adherence, outcomes and adverse events. These temporary measures allowed time for guidance, protective protocols, and risk mitigation measures to be developed (between the Sponsor and trial site) to maximise the continued safety of all trial participants and staff and maintain trial integrity. Taken together, our overall recruitment period was extended by three months to achieve the trial recruitment target. Our primary funder provided additional funding to extend contracts for study staff, as the COVID-19 pandemic directly impeded our trial activities.

Before the COVID-19 pandemic, all clinical trial monitoring activities for EMERGE were conducted in person and on-site. While physical access to our research centre was restricted to patient-facing staff only, the Sponsor team adapted to complete remote monitoring to ensure data and trial integrity and to maintain participant and staff safety. Video-conferencing between the clinical trial monitor and site staff was used to verify the presence of physical documents (e.g. signed informed consent forms) and certified electronic copies of source documents were made available to the trial monitor, as necessary. On-site monitoring visits were re-instated when deemed appropriate and safe from a COVID-19 perspective, in line with Institutional guidance.

## Computer Network Attack

After implementing COVID-19 risk-mitigating strategies, recruitment and follow-up for the EMERGE trial progressively improved ( Figure 1). However, we were further challenged by two independent but successive computer network attacks (CNAs) impacting our Institutions.

First, a national CNA significantly impacted EMERGE through temporary, but significant, limitations on the electronic systems and processes within our hospital. This included the electronic systems used to register patient hospital visits and to report laboratory test results. The clinical trial site staff worked closely with local hospital representatives to develop solutions to ensure appropriate follow-up of trial participants. For example, the approach to laboratory testing was altered: (i) paper-based requests were required; (ii) handwritten completion of patient identifiers onto specimen containers replaced printed stickers; (iii) all specimens were hand-transported to the laboratory; (iv) laboratory staff maintained paper-based records of sample handling; (v) laboratory results were issued as direct printed outputs from relevant laboratory machinery (vs. importing into the clinical laboratory information system); (vi) paper-based results were hand-delivered to the trial site team. This increased workload and delayed results. Recruitment into the EMERGE trial was limited temporarily and we prioritised essential laboratory testing only (i.e. components of our composite primary outcome - week 32 and week 38 glucose levels). When systems returned, all paper-based results were retrospectively entered. Participants safety was not compromised, as metformin required no protocol-mandated safety monitoring.

Second, there was an independent CNA that targeted the EMERGE Trial Sponsor. This led to temporary outages of all electronic systems directly housed and maintained by the Sponsor but did not impact electronic systems maintained by third party providers (e.g. the e-mail system, randomisation, and IMP allocation system). Therefore, there were no interruptions in communication, randomisation or drug dispensing. The EMERGE Electronic Clinical Data Management System (CDMS) was hosted directly by the Sponsor and therefore unavailable during the CNA for site to record new data or for the Sponsor to review live data. Therefore, routine metrics review and reporting was impacted. Importantly, safety reporting and safety data was maintained through a backup paper-based system, and randomisation/IMP dispensing services were uninterrupted. Additional communication and labour-intensive collaboration between the site and Sponsor teams was necessary to monitor additional trial metrics in the short-term.

During the design of the EMERGE trial, appropriate backup systems were established to facilitate interactions between site and the Sponsor, in the event of unavailability of electronic systems. For example, a paper-based backup process facilitated site staff in the expedited reporting of serious adverse events (SAE) to the Sponsor. As email was not impacted, there were no interruptions in communication between the site and the Sponsor, who could receive, review, query, and onward report suspected unexpected severe adverse reactions (SUSAR) to the appropriate Competent Authorities.

To re-instate the CDMS, multiple steps were taken to confirm that there was no impact on the integrity of trial data: (i) review of access logs to confirm that no users accessed the CDMS during the CNA period; (ii) review of modification dates for all electronic case reports forms to confirm that no forms were modified during the CNA period; and (iii) comparison to previous data exports (e.g. for Data Safety and Monitoring Board meetings) to confirming that all data differences were attributable to study staff, occurred before the CNA period, and supported by 100% source data verification. Therefore, we were satisfied that (i) the EMERGE CDMS were not altered or compromised in any way, (ii) the system was unaffected by the CNA, and (iii) the integrity of trial data was maintained throughout. Subsequently, access was re-enabled for the site team, data was entered retrospectively and updated study metrics were available for review by the Sponsor. The CNA impacted the reporting period for the annual Development Safety Update Report (DSUR) to our Competent Authority and Ethics Committee as the CDMS was not accessible on the Development International Birth Date (DIBD). We communicated with our Competent Authority, and the reporting period was extended until the CDMS was accessible and verified and the trial site completed retrospective data entry. The DSUR was compiled and submitted appropriately to the Competent Authority and Ethics Committee.

## Discussion

This manuscript details significant challenges encountered during the conduct of an investigator-initiated, peer-reviewed, funded, academic-sponsored randomised controlled trial in the modern era with impacts from unexpected global events outside the control of the clinical and Sponsor teams. While others have described similar obstacles our trial uniquely faced challenges from both COVID-19 and serial computer network attacks. Many trials were not completed or were terminated early during this period but EMERGE was fully completed in line with our protocol, ICH-GCP and trial regulations, albeit with a time delay.

Our study is not alone in making substantial changes in response to COVID-19. Many trials during this time required protocol amendments for their execution and day-to-day operations. For some, this limited investigators’ ability to collect essential data – e.g. studies measuring where memory and executive function adapted to alternative assessment models including online reviews with a higher risk that participants could receive prompts or memory aids ^12^.

Others describe the limited use of face-to-face contact and increased use of digital healthcare. These changes enabled trials to continue but such changes may limit the development of rapport between patients and site teams ^13, 14^, potentially affecting participant retention. For many, the interactions with researchers are important and benefit the participant directly. During COVID-19, perhaps this dynamic shifted, as individuals with high degrees of healthcare-related anxiety may have appreciated an increased transition to digital and remote healthcare,. Such participants may have been unlikely to participate in the trial if a high degree of in-person interaction between researchers and participants was mandated. Furthermore, COVID-19 highlighted inequity in access to technology across the world. This is termed “digital poverty” and limits patient participation as many global areas do not have access to the tools required to engage in some aspects of healthcare ^15^.

While our trial encompassed women of reproductive age with high technology literacy and reasonable technological access, we faced multiple obstacles. Almost one in five participants were non-native English speakers, and translators were required. While digital translation services are valuable and appreciated by patients, patient satisfaction is higher with face-to-face compared to virtual consultations ^16, 17^; subtleties in patient understanding or body language are more complex to interpret, and health care professionals may be less empathetic or display altered communication styles ^18^. Our trial was also affected by a pause in recruitment from March to June 2020, similar to many other studies ^19^, and an extensive body of work was required to ensure the study could successfully restart ^20^.

COVID-19 was unanticipated and the impact on obstetric care was significant during the antenatal, puerperium and postnatal periods. Women encounter many other pregnant women when attending obstetric care services, in addition to a significant volume of healthcare stuff, thereby increasing their potential exposure to COVID-19. Furthermore, as the pandemic evolved, pregnancy appeared to be a risk factor for more severe COVID-19 including complications such as preterm birth, pre-eclampsia and perinatal death ^21^. Considering these additional risks, it is remarkable that so many women in our jurisdiction remained committed to EMERGE throughout the pandemic and suggests a high degree of comfort with our risk-mitigation strategies and research infrastructure modifications. We could find little evidence of similar studies documenting progress of pregnant women in clinical trials during the pandemic.

The literature confirms that some trials in other patient groups at high risk of COVID-19-related morbidity and mortality (e.g. oncology, respiratory medicine and cardiovascular disease) also continued. A trial of patients following a stroke found that recruitment was difficult, including limited access to technology and redeployment or furloughing of research staff during the trial ^14^. There were high levels of stress in the remaining staff, which likely impacted rapport and relationships with participants. Similarly, a trial of multi-drug resistant tuberculosis (TB) continued but challenged by interruptions in the supply chain, a rapid change to digital reviews, staffing issues and a transition from external central laboratories to local laboratory analyses ^22^. Oncology trials also continued, with modifications. Often the only access to novel oncology therapies is through clinical trials, and it was critically important that this access was maintained for individuals with cancer diagnoses, despite the pandemic. Although some healthcare professionals believed that clinical trials were high-risk to these patients others importantly and appropriately advocated for informed participant choice, including regularly reminding participants about the option to withdraw consent from an ongoing trial ^23^. Data show that there were higher levels of hesitation and reluctance in some patient groups related to COVID-19 ^24^. Although there were significant impacts, it is reassuring to see those patients within our trial, and other trials, remained interested and committed to clinical trials and that progress continued to be made for high-risk patient groups.

Cardiovascular trials were significantly impacted by the pandemic due to the increased susceptibility of the patient population to severe COVID-19 and shielding protocols. The European Society of Cardiology issued a consensus statement outlining key recommendations for conducting trials ^25^ including practical solutions including limiting in-person visits, minimising visit duration, ensuring that trial participants did not interact in-person; and increased use of telephone/video-conferencing and contact tracing logs. We implemented local solutions similar to these recommendations within EMERGE, including adjustments necessary for the obstetric nature of our trial in that each trial participant incorporated two individuals (mother and baby) with different healthcare specialties and systems.

We remained committed to the conduct of EMERGE throughout the pandemic as we remained committed to the rationale and underlying ethical considerations. Initially, pregnant women were not considered particularly vulnerable or high-risk for COVID-19; therefore, the risk-benefit analysis for the conduct of EMERGE remained in favour of the trial. As the pandemic progressed and recruitment resumed, we were confident in our risk mitigation strategies and participants continued to enter the trial and complete trial activities. Participant safety and trial integrity were entirely maintained throughout the trial. No COVID-19 transmission occurred between site teams and participants. As the COVID-19 vaccination program rolled out, research staff were vaccinated early and pregnant women in Ireland were advised to attend for vaccination; this further increased our confidence in continuing with EMERGE. Our participants remained committed to EMERGE throughout the pandemic, consistent with the social contract between them and the EMERGE research team. Whilst we recorded cases of COVID-19 in EMERGE participants as adverse events, we did not think it necessary to incorporate COVID-19 as a trial outcome.

 Taken together, we adapted the EMERGE trial during the COVID-19 pandemic to incorporate elements of decentralised clinical trials (DCT), as we increased accessibility and reduced participant burden to attend the clinical trial site ^26^ and prioritising the needs and care of the patient ^27^. Although DCT classically reduce workload for the site team ^28^, the unique requirements of COVID-19 did not confer this particular advantage on EMERGE, although it did successfully minimise patient risk ^29– 31^. For future trials, our group would be more comfortable employing additional features of DCT from the outset, with an increased use of telemedicine and/or home visits, if appropriate and acceptable to our study population ^31^. The contribution of public and patient health carers in the design of future trials considering DCT methodologies is essential to this further.

To our knowledge, this is the first paper to comment on the effect of computer network attacks on the execution and conduct of a regulated, randomised, controlled IMP trial, particularly in a vulnerable population. Although we are aware of other studies within Europe that have been affected by these types of attacks, we are the first to outline our approach in the hope that others (particularly other Academic Institutions that Sponsor clinical trials) can learn from our unfortunate experience and implement additional risk mitigating strategies in the design phase of future clinical trials.

## Conclusion

In summary, we faced several complex and diverse challenges during the EMERGE trial. Overcoming these obstacles required successful teamwork, communication, motivation, innovation, and adaptability from staff and sponsor personnel, as well as flexibility and resilience from our patient cohort during a particularly stressful time. The lessons learned from EMERGE have strengthened our research infrastructure and up-skilled staff for future trials, including an increased awareness of key elements of DCTs.

## Data Availability

Figshare. Repository for EMERGE Covid paper ^32^ This project contains the following underlying data Supplementary data (Outline of the semi-structured interview) Figures PIL and consent https://doi.org/10.6084/m9.figshare.30018712.v1 Data are available under the terms of the Creative Commons Attribution 4.0 International Public License (CC BY 4.0).
