# Supplementary material for: Navigating Complexities In Randomized Trials: Insights From The EMERGE Trial Amidst Pandemic And Cybersecurity Threat
Source: HRB Open Res. 2025 Sep 10;8:101. [Version 1] doi: 10.12688/hrbopenres.14233.1 (PMC12784043; doi:10.12688/hrbopenres.14233.1)
Supplement: Peer review history

# Reviewer report: Reviewer response for version 1

**DOI:** 10.21956/hrbopenres.15654.r52057

**Published:** 2026-01-08

**Authors:** Shahazad Niwazi Qurashi

**Affiliations:** 1 Jazan University, Jizan, Saudi Arabia

This article is a **useful and timely reflection** on the operational realities of conducting randomized trials during global crises. It contributes meaningfully to the literature on trial resilience, digital security, and adaptive research management. While not methodologically exhaustive, it offers **practical lessons** that can inform future trial planning and crisis preparedness.

Is the work clearly and accurately presented and does it cite the current literature?

Yes

If applicable, is the statistical analysis and its interpretation appropriate?

I cannot comment. A qualified statistician is required.

Are all the source data underlying the results available to ensure full reproducibility?

Yes

Is the study design appropriate and is the work technically sound?

Yes

Are the conclusions drawn adequately supported by the results?

Yes

Are sufficient details of methods and analysis provided to allow replication by others?

Yes

Reviewer Expertise:

Health Informatics, Disease data analysis, Cyber Security, and AI

I confirm that I have read this submission and believe that I have an appropriate level of expertise to confirm that it is of an acceptable scientific standard.

# Reviewer report: Reviewer response for version 1

**DOI:** 10.21956/hrbopenres.15654.r50229

**Published:** 2025-10-07

**Authors:** C.V.Guru Rao

**Affiliations:** 1 COMPUTER SCIENCE AND ENGINEERING, Gayatri Vidya Parishad College of Engineering (Ringgold ID: 212009), VISHAKAPATNAM, Andhra Pradesh, India

1. The references must be updated before submitting for a camera-ready manuscript.

 2. An elaborated survey will arrive at the exact problem specified for this research.

 3. Methods explained can be further described in terms of explanation.

 4. Results presented must be explained in detail.

 5. Good to revise the conclusion in terms of research objectives vis-à-vis research contributions, comparison of your approach with the existing state of the art techniques in the literature, SWOT Analysis of your solution, and applicability of your research outcome, future scope of research on this topic.

Is the work clearly and accurately presented and does it cite the current literature?

Yes

If applicable, is the statistical analysis and its interpretation appropriate?

Not applicable

Are all the source data underlying the results available to ensure full reproducibility?

Yes

Is the study design appropriate and is the work technically sound?

Yes

Are the conclusions drawn adequately supported by the results?

Yes

Are sufficient details of methods and analysis provided to allow replication by others?

Yes

Reviewer Expertise:

Software Engineering, Embedded Systems-on-Chip Design and Testing, Networks and Security, Cyber Security, Cloud Security, Health Care Sistems etc

I confirm that I have read this submission and believe that I have an appropriate level of expertise to confirm that it is of an acceptable scientific standard.
